# Prevalence of bisphosphonate associated osteonecrosis of the jaws in multiple myeloma patients

**DOI:** 10.1186/1746-160X-6-11

**Published:** 2010-07-08

**Authors:** Christian Walter, Bilal Al-Nawas, Norbert Frickhofen, Heinold Gamm, Joachim Beck, Laura Reinsch, Christina Blum, Knut A Grötz, Wilfried Wagner

**Affiliations:** 1 Klinik für Mund-, Kiefer- und Gesichtschirurgie, Johannes Gutenberg-Universität Mainz; Augustusplatz 2, 55131 Mainz, Deutschland; 2 Klinik Innere Medizin III, Dr. Horst Schmidt Klinik Wiesbaden, Ludwig-Erhard-Str. 100, 65199 Wiesbaden, Deutschland; 3 III Medizinische Klink und Poliklinik, Johannes Gutenberg-Universität Mainz, Langenbeckstr. 1, 55131 Mainz, Deutschland; 4 Klinik für Mund-, Kiefer- und Gesichtschirurgie, Dr. Horst Schmidt Klinik Wiesbaden, Ludwig-Erhard-Str. 100, 65199 Wiesbaden, Deutschland

## Abstract

**Background:**

Bisphosphonate-associated osteonecrosis of the jaws (BP-ONJ) is an adverse effect of bisphosphonate treatment with varying reported incidence rates.

**Methods:**

In two neighboring German cities, prevalence and additional factors of the development of BP-ONJ in multiple myeloma patients with bisphosphonates therapy were recorded using a retrospective (RS) and cross-sectional study (CSS) design. For the RS, all patients treated from Jan. 2000 - Feb. 2006 were contacted by letter. In the CSS, all patients treated from Oct. 2006 - Mar. 2008 had a physical and dental examination. Additionally, a literature review was conducted to evaluate all articles reporting on BP-ONJ prevalence. PubMed search terms were: bisphosphonat, diphosphonate, osteonecrosis, prevalence and incidence.

**Results:**

In the RS, data from 81 of 161 patients could be obtained; four patients (4.9%) developed BP-ONJ. In the CSS, 16 of 78 patients (20.5%) developed BP-ONJ. All patients with BP-ONJ had received zoledronate; 12 of these had had additional bisphosphonates. All except one had an additional trigger factor (tooth extraction [n = 14], dental surgical procedure [n = 2], sharp mylohyoid ridge [n = 3]).

**Conclusion:**

The prevalence of BP-ONJ may have been underestimated to date. The oral examination of all patients in this CSS might explain the higher prevalence, since even early asymptomatic stages of BP-ONJ and previously unnoticed symptomatic BP-ONJ were recorded. Since nearly all patients with BP-ONJ had an additional trigger factor, oral hygiene and dental care might help to reduce BP-ONJ incidence.

## Background

Multiple myeloma is characterized by neoplastic proliferation of plasma cells clones. The most frequent complications are anemia, renal failure, recurrent bacterial infections, pathological fractures, and hypercalcemia due to tumor-induced bone destruction via osteoclastic bone resorption [1,2].

Bisphosphonates are used in the treatment of multiple myeloma because of their inhibitory effect on osteoclastic activity. Depending on the ligands, bisphosphonates are grouped into nitrogen-containing and non-nitrogen-containing bisphosphonates. The former inhibit the mevalonate pathway, and the latter are build into the ATP-molecule; both result in cytotoxic effects to the osteoclast [3,4].

Bisphosphonates have a beneficial effect on pain and can reduce fractures in multiple myeloma patients, [5] thereby increasing quality of life for the patient.

Adverse effects can be separated into four groups: acute-phase-reactions, upper aerodigestive tract issues and effects concerning renal function [6]. In 2003, the fourth adverse effect, bisphosphonate associated osteonecrosis of the jaw (BP-ONJ), was described for the first time [7] and has been subsequently diagnosed with increasing frequency [8].

At first, a causal connection between BP-ONJ and bisphosphonates was denied on the grounds that the patients had additional risk factors for osteonecrosis that could explain the higher incidence (cancer, radiation or chemotherapy, special medications such as steroids, infections of dental or sinus origin, dental procedures, anemia or local anesthetics with vasoconstrictors) [9]. In the meantime, BP-ONJ has been included in the summary of product characteristics. There is evidence that the presence of several of these risk factors is related to an increased risk of developing a BP-ONJ [10].

According to the American Association of Oral- and Maxillofacial Surgeons (AAOMS), BP-ONJ is defined as the presence of exposed, necrotic bone in the maxillofacial region that has persisted for more than eight weeks in a patient with current or previous bisphosphonate therapy and no history of radiation to the jaws [11]. Patients present with exposed bone, and fistulas to the oral cavity or external skin. If there is additional infection in the bone or soft tissues, abscesses, osteolysis and pathological fractures may result. Several theories concerning etiology and pathogenesis are currently being discussed in addition to the suppressed bone turnover theory [12]. Of particular interest are the antiangiogenic effects of bisphosphonates and the possible subsequent development of avascular osteonecrosis [13], as well as the adverse impact on the mucosal layer covering the bone via a proapoptotic effect on keratinocytes [14]. Common potential trigger factors for the condition have been identified, the most frequent being a recent dental surgical procedure such as a tooth extraction. Other seemingly significant factors include periodontal disease with odontoseisis and denture pressure sores [8]. Therapy of BP-ONJ is difficult and ranges from simple mouth rinses for asymptomatic exposed bone, to debridement and huge resections of the mandible or of the maxilla, the latter having a large impact on the quality of life for effected patients.

The incidence of the BP-ONJ in multiple myeloma patients is largely unknown; to date, only a few studies and one web based survey have been published (see table 1). In most studies, only the patients who reported symptoms received an oral exam, rather than all patients at risk for the condition, thereby potentially underestimating the magnitude of this side effect.

**Table 1:** Published incidence studies.

Year	Author	Study design	All patients orally examined	Disease	Patients (n)	BP-ONJ cases	Incidence (%)	Used bisphosphonates in BP-ONJ patients
2005	Bamias [24]	pros	no	breast ca	70	2	3	Z, PZ, ZI
				mult myel	111	11	10	
				prostate ca	46	3	7	
	
	Durie [25]	web survey	no	mult myel	904	62	6.9	Z,P
				breast ca	299	13	4.3	
	
	Guarneri [26]	retro	no	breast ca	48	3	6	P
	
	Maerevoet [27]	retro	no	mult myel	194	9	5	P, Z
				breast ca				

2006	Badros [28]	retro	yes	mult myel	340	11	3.2	P, Z, PZ
	
	Calvo-Villas [29]	retro	no	mult myel	64	7	11	Z
	
	Dimopoulos [30]	pros	unclear	mult myel	202	15	7.4	Z, P, ZP, ZI
	
	Hoff [31]	retro	no	breast ca	1338	16	1.2	P, Z, PZ
				mult myel	448	14	3.1	
	
	Sanna [32]	pros	yes	breast ca	81	5	6	P, Z
	
	Tosi [33]	retro	no	mult myel	259	9	3.5	Z
	
	Zervas [34]	pros	no	mult myel	254	28	11.0	Z, P, ZP

2007	Aguiar Bujanda [35]	css	yes	breast ca	35	4	11	Z
	
	Corso [36]	retro	no	mult myel	106	8	8	Z, PZ
	
	García Sáenz [37]	pros	no	prostate ca	104	3	3	Z
	
	Jadu [38]	retro	yes	mult myel	655	21	3.2	P
	
	Mavrokki [39]	estimation	no	malignancies			0.9-1.2	
	
	Ortega [40]	retro	no	prostate ca	52	6	12	Z
	
	Petrucci [41]	unclear	no	mult myel	311	22	7.1	P, PZ, Z
	
	Wang [15]	retro	no	mult myel	292	11	3.8	Z, P, PZ
				breast ca	81	2	3	
				prostate ca	69	2	3	
	
	Wilkinson [42]	retro	no	malignancies	14 349		5.48	Z, P, PZ

2008	Boonyapakorn [22]	pros	yes	mult myel	58	10	17	P, PZ, IZ, Z
	
	Fehm [43]	retro	no	breast ca	233	10	4.3	Z, ICPZ
	
	Ibrahim [44]	retro	no	breast ca	220	5	2.3	PZ, Z
				mult myel	59	2	3	
	
	Walter [23]	css	yes	prostate ca	43	8	19	IZ, PZ, Z

2009	Walter [45]	retro	no	breast ca	75	4	5.3	Z, PZI
	
	this retrospective study	retro	no	mult myel	81	4	4.9	U, PZ
	
	this cross-sectional study	css	yes	mult myel	78	16	21	Z, PZ, IZ, PZI

All published studies with BP-ONJ incidences compared to the results of these two studies in the last two lines.

Abbreviations: n, number of patients; BP-ONJ, bisphosphonate-associated osteonecrosis of the jaws; css, cross-sectional study; retro, retrospective study; ca, cancer; mult myel, multiple myeloma; stage I-III, Durie [16] classification; Z, zoledronate; P, pamidronate; I, ibandronate

We conducted two studies in two midsize neighboring German cities. The aim was to evaluate the prevalence of BP-ONJ in multiple myeloma patients using two different study designs and to compare this with existing published data. Further aims were to detect additional factors that may be related to the development of BP-ONJ.

## Methods

Two studies with two different populations were conducted: one retrospective study and one cross sectional study. Inclusion criteria for both studies were a diagnosis of multiple myeloma and bisphosphonate therapy; the duration of bisphosphonate therapy was not taken into account. A BP-ONJ case was defined as exposed necrotic bone existing over a time span of at least eight weeks, with no radiation of the head and neck area in the patient's history [11,15].

The retrospective study was conducted in the Department of Internal Medicine (Oncology, Hematology and Palliative Medicine) at the Dr. Horst Schmidt Klinik Wiesbaden, Germany. Due to an in-house cancer registry, all patients treated in this clinic suffering from multiple myeloma are registered. In the study time span from January 2000 to February 2006, 161 patients with multiple myeloma and bisphosphonate treatment were registered. All patients were contacted by letter and/or phone; if contact information was available for their physicians and dentists, they were contacted as well. In the end, we obtained data for 81 patients (50.3%). Additional diseases, medication and risk factors for osteonecrosis were recorded from patient histories.

The second study had a cross sectional design and was conducted in the Department of Haematology and Oncology at the Johannes Gutenberg-University Mainz, Germany. All 78 patients with multiple myeloma treated from October 2006 to March 2008, regardless of the date of diagnosis, were comprised. The same data elements were collected as in the retrospective study, but patients in the cross sectional study also underwent an additional examination (dental and medical) and missing data were completed. In the oral examination conducted by a dentist, the patients were checked for oral hygiene (visible existence of dental plaque and tartar using the Silness-Loe plaque index), signs of inflammation, damages to the oral mucosa and exposed bone.

The protocol was approved by the local ethics committee: 837.099.06 (5197). Informed consent was obtained for each patient. No funding was received for this study.

In addition to a descriptive analysis, p-values were calculated with the Mann-Whitney-Test, the Fisher's exact test and the Pearson's Chi-Quadrate-Test. Statistical analysis was conducted using SPSS 16 for Windows (SPSS Inc. Headquarters, Chicago, Illinois).

In addition, Pubmed was queried for literature on BP-ONJ occurrence. The search was done in December, 2008 using the following search terms: bisphosphonate, diphosphonate, incidence and prevalence. All papers reporting on incidence or prevalence of BP-ONJ were analysed and are reported in Table 1.

## Results

### Retrospective study

161 patients with multiple myeloma and bisphosphonate treatment were identified. Data for 81 patients (50%) could be obtained, 69 had passed away, and 11 either refused participation or we failed to locate them. The 81 recruited patients (36 women and 45 men) had an average age of 69.8 years (range 44 - 95 years); the average age at the time of multiple myeloma diagnosis was 63.7 years (range 40 - 83 years). All administered bisphosphonates were nitrogen-containing bisphosphonates. Patients had been treated for a mean time of 48 months (range 1 - 204). 3% of the patients had received alendronate (70 mg) per os, 34% had received zoledronate, 26% pamidronate and 15% ibandronate. For the remaining 12%, the bisphosphonate therapy had varied, so that they had received different bisphosphonates in the course of their treatment (combinations were: pamidronate/zoledronate, ibandronate/zoledronate and ibandronate/pamidronate/zoledronate).

Altogether, four patients (5%), three men and one women aged 53, 71, 74 and 65 respectively, suffered from bisphosphonate associated osteonecrosis of the jaw. Two of these patients had only received zoledronate; the other two patients had been treated with pamidronate followed by zoledronate for an average duration of 64 months (range 27 - 128 months). All patients who developed BP-ONJ in the mandible did so after a tooth had been removed. All patients with BP-ONJ had been treated with cytotoxic drugs in addition to bisphosphonates; only one patient with BP-ONJ was receiving cytotoxic drugs at the time of BP-ONJ diagnosis. In comparison, 82% of all patients without BP-ONJ had had chemotherapy. None of the patients with BP-ONJ smoked, although 8 patients without BP-ONJ did. 8 patients suffered from diabetes mellitus but none of them developed BP-ONJ; 9 patients had further malignant diseases and one developed BP-ONJ.

### Cross sectional study

78 patients (47 men and 31 women) with multiple myeloma and current bisphosphonate therapy with an average age of 63.5 years (range 53 - 79 years) at oral examination had been treated in the specified time span of 10/06 to 03/08 in the Department of Haematology and Oncology of the Johannes Gutenberg-University Mainz, Germany (Table 2). The average age at the time of multiple myeloma diagnosis was 59.8 years (range: 41 -84 years).

**Table 2:** Data of the cross sectional study.

	All patients	Patients with BP-ONJ	Patients without BP-ONJ	Difference
Number (percentage)	78 (100%)	16 (21%)	62 (80%)	

Age at examination (SD)	63.5 (10.1)	61.9 (21.0)	64.1 (10.5)	p = 0.18

Age at MM diagnosis	59.8 (11.1)	54.1 (8.8)	61.3 (11.2)	p = 0.02

Men	47	9	38	p = 0.78
	
Women	31	7	24	

MM stage I	17 (22%)	3 (4%)	14 (18%)	
	
MM stage II	13 (17%)	2 (3%)	11 (14%)	p = 0.79
	
MM stage III	48 (62%)	11 (14%)	37 (47%)	

Bisphosphonate infusions	28.1	48.4 (range 9 - 111)	23.3 (range 1 - 104)	p < 0.001

Zoledronate				
Patients	49 (63%)	4 (5%)	45 (58%)	
Infusions	15	21	15	
	
Pamidronate + zoledronate				
Patients (percentage)	20 (26%)	9 (12%)	11 (14%)	
infusions	33, 22	26, 30	39, 15	
	
Ibandronate + zoledronate				p = 0.001
Patients (percentage)	6 (8%)	1 (1%)	5 (6%)	
infusions	6, 15	4, 28	7, 12	
	
Pamidronate + zoledronate + ibandroante				
Patients (percentage)	3 (4%)	2 (3%)	1 (%)	
infusions	38, 20, 5	27, 28, 3	59, 5, 10	

Corticosteroids	59 (76%)	13 (17%)	46 (61%)	p = 0.75

Chemotherapy	64 (82%)	13 (17%)	51 (67%)	p = 1.0

Diabetes mellitus	6 (8%)	2 (3%)	4 (5%)	p = 0.6

Further malignant diseases	3 (4%)	0	3 (4%)	p = 1.0

Smoker	8 (10%)	1 (1%)	7 (9%)	p = 1.0

Patients with all teeth in situ	8 (10%)	0	8 (10%)	p = 0.2

Caries	16 (21%)	2 (3%)	14 (18%)	p = 0.50

Denture	37 (47%)	9 (12%)	28 (36%)	p = 0.58

P-values were calculated with the Mann-Whitney-Test and the Fisher's exact test and the Pearsons's Chi-Quadrate-Test. Abbreviations: BP-ONJ, bisphosphonate associated osteonecrosis of the jaws; SD, standard deviation; MM, multiple myeloma; +, sequent

According to the multiple myeloma classification of Durie and Salmon [16], 17 patients were in stage I, 13 in stage II and 48 patients in stage III. 49 patients had received only zoledronate, 20 patients had previously taken pamidronate before receiving zoledronate, six patients had first received ibandronate followed by zoledronate, and three patients had received pamidronate, zoledronate and ibandroante sequentially. 59 patients (76%) had been given corticosteroids during their course of therapy, and 64 patients (82%) had received chemotherapy (melphalane, idarubicine, cyclophosphamide, busulfane, bortezomib, thalidomide, etoposide).

All together, 16 patients (21%) developed BP-ONJ. These patients had received an average of 48.4 bisphosphonate infusions compared to 23.3 infusions in patients without BP-ONJ. Four of these patients had had only zoledronate and nine patients had received pamidronate and zoledronate sequentially; one patient had first received ibandronate followed by zoledronate; two patients had pamidronate, zoledronate and ibandronate sequentially. In 5 patients, BP-ONJ was located in the mandible, in another 5 patients in the maxilla, and in 6 patients in both jaws. Ten patients had previously had a tooth extraction at the side of the BP-ONJ; two further patients had recently undegone a dental surgical procedure, and three patients developed BP-ONJ at the mylohyoid ridge. In one patient, no possible trigger factor for ONJ was obvious. 13 of the 16 BO-ONJ patients had undergone additional chemotherapy and steroids. Three of these patients had received chemotherapy one or two months before the BP-ONJ diagnosis. In 10 patients, chemotherapy had been applied more than 12 months before. Two patients with and four patients without BP-ONJ additionally suffered from diabetes mellitus. The entire group of patients had 9 smokers and 1 developed BP-ONJ. Three patients without BP-ONJ suffered from an additional malignant tumor.

## Discussion

The aim of these two studies was to evaluate the prevalence of BP-ONJ in patients with multiple myeloma and bisphosphonate therapy.

In the retrospective study, the average age of the patients at the time of multiple myeloma diagnosis was 63.7 (SD: 10.9) years. In the cross-sectional study the age was 59.8 (SD: 11.1) years. Both are marginally younger than that described in the cancer register in Saarland, Germany, [17] with a peak at the 65-75 year age range and a median age of disease diagnosis at the range of 60 to 64 years. The median age for multiple myeloma patients in the United States is 70 years [18]. 4 out of 81 patients (5%) in the retrospective study and 16 out of 78 (21%) in the cross sectional study had BP-ONJ.

In both study populations, zoledronate was the bisphosphonate most often used. In the cross sectional study, all patients had received either zoledronate exclusively, or zoledronate in sequence with other bisphosphonates; the same is true for 32 out of 72 patients in the retrospective study. A critical point for the retrospective study design might be the lack of detection of asymptomatic BP-ONJ especially as no staging of the disease can be done with this design. In both studies, all patients who developed BP-ONJ had received zoledronate at some point during the course of treatment. The association between zoledronate and BP-ONJ is explained by the higher potency of zoledronate as compared to the other bisphosphonates, as well as zoledronate's wide spread use among cancer patients. Except for one patient in the cross sectional study, all patients showed additional trigger factors such as tooth extractions (n = 14), dental surgical procedure (n = 2) or a sharp mylohyoid ridge (n = 3), a spot with a very thin mucous membrane. No further trigger factor could be detected for only one patient with a necrosis in the maxilla and the mandible. Chemotherapy did not show an influence on BP-ONJ prevalence in this study. It should however be noted that the chemotherapeutics used were heterogenous and did not allow for a further subgroup analysis.

Considering the high percentage of cases with an identifiable trigger factor, a sensible prevention tactic may be to refer patients with planned bisphosphonate therapy to a dentist or an oral and maxillofacial surgeon who is familiar with this kind of disease. This could be analogous to the management of patients who are about to receive head and neck radiation [19]. Non-restorable teeth could be extracted and restorations could be done. After commencement of bisphosphonates therapy, improved oral hygiene could prevent further dental surgical procedures such as tooth extractions. Additionally, even currently asymptomatic exposed necrotic bone might be prevented from becoming infected. A decrease in BP-ONJ among patients involved in such a preventive program has recently been described [20].

Across both studies, the most affected site was the mandible (9 patients). Six patients had an osteonecrosis in the mandible as well as in the maxilla, and five patients had the osteonecrosis in the maxilla only. This skewed distribution might be explained by the reduced blood supply in the mandible as compared to the maxilla [21].

All incidence and prevalence studies that have been published are shown in Table 1. One problem of at least this retrospective study is the high rate of patients that was not included in the study and could therefore be a selection bias. Especially these patients might have had a severe course of disease and co-morbidities involving further medications that might be associated with a greater odd to develop an osteonecrosis. There are more reasons that may explain the higher prevalence found in this cross sectional study as compared to the studies in table 1, the largest of which may be the study design itself. Due to the retrospective design of most other studies, complete oral exams of patients to look for BOJ have rarely been done. Only Boonyapakorn [22] for multiple myeloma patients and Walter [23] for prostate cancer patients describe similarly high findings with a similar study design. Medical examinations alone may not be able to detect all cases of BOJ, particularly those cases of early stage BP-ONJ with asymptomatic exposed necrotic bone. Dental examinations that focus on BP-ONJ can detect a barely visible osteonecrosis, for example on the mylohyoid ridge, and may therefore explain the higher incidences in these studies. Although there is no statistically significant difference, the prevalence for all retrospective studies for multiple myeloma ranges from 3 to 11% and for prospective studies from 7 to, including this study, 21% (p = 0.06). In studies with such small numbers of patients, the detection or non detection of a BP-ONJ has a huge influence on the resulting prevalence or incidence.

## Conclusion

The prevalence of BP-ONJ may have been underestimated to date. The oral examination of all patients in this CSS might explain the higher prevalence, since even early asymptomatic stages of BP-ONJ and previously unnoticed, symptomatic BP-ONJ were recorded. Since nearly all patients with BP-ONJ had an additional trigger factor, oral hygiene and dental care might help to reduce BP-ONJ incidence.

## Competing interests

CW: received research funding from Norvartis for another project and gave speeches for Roche

BA: gave lectures for Roche

NF: no conflict of interest

HG: no conflict of interest

JB: no conflict of interest

LR: no conflict of interest

CB: no conflict of interest

KAG: gave speeches for Roche and Novartis

WW: no conflict of interest

## Authors' contributions

CW: Design, data collection, analysis, interpretation, paper written

BA: Design, analysis, interpretation, approval of paper

NF: Design, data collection, analysis, interpretation, approval of paper

HG: Design, data collection, analysis, interpretation, approval of paper

JB: Design, data collection, approval of paper

LR: Design, data collection, analysis, interpretation, approval of paper

CB: Design, data collection, analysis, interpretation, approval of paper

KAG: Design, data collection, analysis, interpretation, approval of paper

WWr: Design, analysis, interpretation, approval of paper
